# *miR-100-5p* inhibition induces apoptosis in dormant prostate cancer cells and prevents the emergence of castration-resistant prostate cancer

**DOI:** 10.1038/s41598-017-03731-8

**Published:** 2017-06-22

**Authors:** Noushin Nabavi, Nur Ridzwan Nur Saidy, Erik Venalainen, Anne Haegert, Abhijit Parolia, Hui Xue, Yuwei Wang, Rebecca Wu, Xin Dong, Colin Collins, Francesco Crea, Yuzhuo Wang

**Affiliations:** 1 Honors Biotechnology Program, Department of Microbiology and Immunology, University of British Columbia, 2329 West Mall, Vancouver, BC V6T 1Z4 Canada; 2 Experimental Therapeutics, BC Cancer Research Centre, Vancouver, BC Canada; 3 The Vancouver Prostate Centre, Vancouver General Hospital, Vancouver, BC Canada; 4 School of Life, Health and Chemical Sciences, The Open University, Milton Keynes, UK; 5 Department of Urologic Sciences, University of British Columbia, Vancouver, BC Canada

## Abstract

Carcinoma of the prostate is the most common cancer in men. Treatment of aggressive prostate cancer involves a regiment of radical prostectomy, radiation therapy, chemotherapy and hormonal therapy. Despite significant improvements in the last decade, the treatment of prostate cancer remains unsatisfactory, because a significant fraction of prostate cancers develop resistance to multiple treatments and become incurable. This prompts an urgent need to investigate the molecular mechanisms underlying the evolution of therapy-induced resistance of prostate cancer either in the form of castration-resistant prostate cancer (CRPC) or transdifferentiated neuroendocrine prostate cancer (NEPC). By analyzing micro-RNA expression profiles in a set of patient-derived prostate cancer xenograft tumor lines, we identified miR-100-5p as one of the key molecular components in the initiation and evolution of androgen ablation therapy resistance in prostate cancer. *In vitro* results showed that miR-100-5p is required for hormone-independent survival and proliferation of prostate cancer cells post androgen ablation. In Silico target predictions revealed that miR-100-5p target genes are involved in key aspects of cancer progression, and are associated with clinical outcome. Our results suggest that mir-100-5p is a possible therapeutic target involved in prostate cancer progression and relapse post androgen ablation therapy.

## Introduction

Current cancer therapeutics are often effective in treating clinically evident tumors; however, patient mortality is often due to the permanence of residual tumor cells (RTCs) or disseminated tumor cells (DTCs) that are highly resistant to therapy and capable of generating metastatic and incurable diseases^1^. These deadly seeds are able to remain “dormant”, escaping detection, for an extensive period of time until the emergence of a clinically evident disease. The presence of dormant cancer cells (DCCs) has been described as early as the 1930s; however, interest in dormancy only started gaining momentum by the end of the 20^th^ century. The prevalent model describes arrested DCCs escaping conventional therapeutic pressure that only target rapidly dividing cells^2^. Proposed therapeutic strategies in targeting DCCs include inducing or maintaining dormancy and targeting survival mechanisms^2^.

Androgen-deprivation therapy (ADT) is often used to treat high risk localized prostate cancer (PCa) and to prevent the progression towards metastasis^3^. ADT-treated PCa, however, often regress to a dormant state that can last for a variable number of years. ADT eventually becomes ineffective due to the emergence of clinically evident castration (Cx)-resistant PCa (CRPC) or transdifferentiated neuroendocrine PCa (NEPC). While there are significant genetic overlaps between CRPC and NEPC manifestations of PCa resistance, the former is dependent of androgen receptor activation while the latter is androgen indifferent^4^. Current clinical treatments for both CRPC and NEPC only extend patient survival time by a few months and are characterized by progressively shorter remission times. Targeting ADT-induced DCCs may be the key to preventing additional cascades of dormancy-relapse cycles and the emergence of CRPC and/or NEPC. However, little is known about the molecular mechanisms underpinning the emergence of ADT-induced DCCs.

Efforts at further characterizing the cellular mechanisms underpinning cancer dormancy have reached a significant bottleneck due to inadequate experimental models. Most models for cancer dormancy involve the enrichment of quiescent and drug resistant clones^5–7^. Further, few *in vivo* models, if any, truly capture the phenomenon in which DCCs can predictably give rise to treatment resistance and clinically evident relapse.

We have previously proposed that an epigenetic-noncoding interactome may be responsible for the plasticity of dormant cancer cells^8, 9^. In a highly inter-connected network, long non-coding RNAs, microRNAs (miRNAs), and epigenetic effectors orchestrate key cellular functions to facilitate the stochastic needs of neoplastic cells. miRNAs are short non-coding RNA molecules (~22 nucleotides long) that function in post-transcriptional gene regulation by binding to the 3′ untranslated region of the targeted mRNA and obstructing resultant gene expression^10^. miRNAs are deeply involved in key cellular functions such as cell proliferation, apoptosis, metabolism, and their de-regulation has been frequently linked to carcinogenesis^11, 12^. Dysregulated miRNAs were first reported in cancer in 2002 in chronic lymphocytic leukemia (CLL) and ever since, tumor miRNA profiles have been used to define relevant disease subtypes, patient survival, and treatment response^13^. Interestingly, miRNAs target more than 60% of known human mRNAs^14^ and are able to inhibit gene expression through highly reversible mechanisms^15–17^. With emerging studies, miRNAs are rapidly developing as attractive candidates for governing the plasticity of DCCs. To the best of our knowledge, however, miRNAs have not yet been studied in ADT-induced PCa dormancy.

We have recently developed a unique panel of patient-derived xenograft (PDX) tumor lines in mouse models that closely recapitulates the clinical progression of PCa post androgen ablation through host castration^18–20^. These high fidelity PCa models based on sub-renal capsule implantation have proven valuable for cancer research since they retain key biological properties of the original malignancies, including histopathology, genomic profile and metastatic ability^21^. In this study, we have employed representative PDX models that mimic ADT-induced dormancy or relapse in the form of CRPC or NEPC. We aimed to investigate the involvement of miRNAs in the initiation and progression of PCa resistance to ADT.

## Results

### Castration-induced PCa dormancy and resistance is recapitulated in PDX models

**Figure 1:**
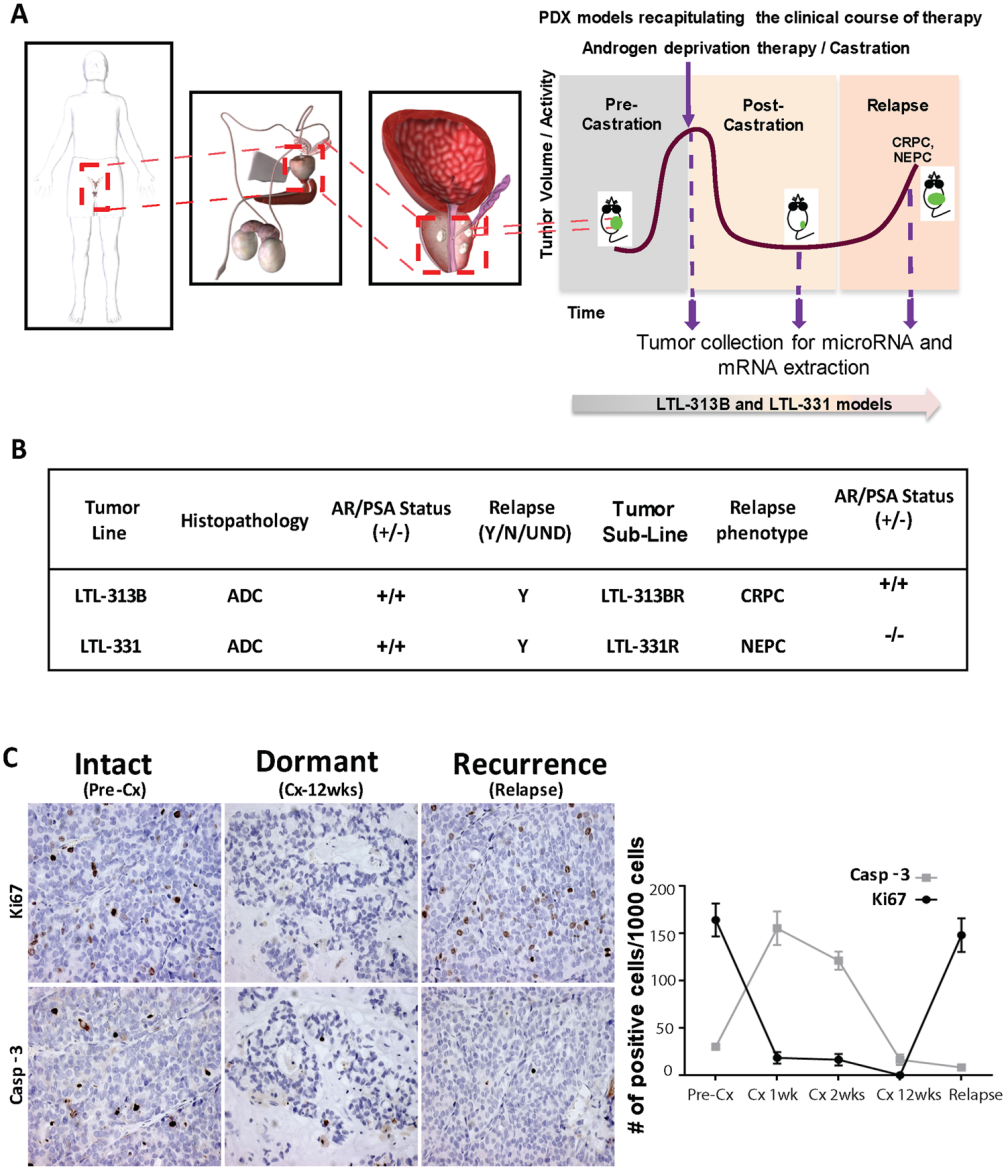
PDX models recapitulating PCa disease progression. PDX models recapitulating clinical course of therapy: (**A**) Two hormone-sensitive prostate PDX models were generated and subjected to surgical castration (androgen deprivation). Upon the emergence of a castration-resistant sub-line, pathological examination stratified the tumors into either adenocarcinoma (CRPC) or neuroendocrine (NEPC) phenotypes. Drawings are adapted from BioDigital Human Application (human.biodigital.com) who hold the copyright and permit this publication under an Open Access license. (**B**) Characterization of the fate of PDX models (abbreviations: LTL = living tumor laboratory, ADC = adenocarcinoma, Y = yes, N = no, UND = undetermined, CRPC = castration-resistant prostate cancer, NEPC = neuroendocrine prostate cancer). Evidence of therapy induced dormancy and resistance. (**C**) Surgical castration can induce tumor dormancy *in vivo* using PCa PDX models: (**A**) Immunohistochemical staining of tissues obtained from LTL-313B PDX model. Cell proliferation and apoptosis are detected with Ki67 and Caspase-3 antibodies measuring protein expression, respectively. (**D**) Quantification of proliferation and apoptosis in LTL-313B tumors through determining the number of positively stained cells/1000 cells in the pre-castration, dormancy and relapse phases.

PCa PDX models from the Living Tumor Laboratory (LTL; http://www.livingtumorcentre.com/) repository were employed to develop and characterize ADT-induced dormancy (Fig. 1A). Specifically, hormone-sensitive PCa tumor lines were subjected to surgical Cx (androgen deprivation) in an adapted protocol by Lin *et al*.^21^ Upon the emergence of a Cx-resistant sub-line, pathological examination was employed to distinguish between the adenocarcinoma (CRPC) and neuroendocrine (NEPC) phenotypes (Fig. 1B and Supplementary Figure S1A). To date, 7 out of 11 models relapsed as an androgen-independent tumor sub-line while the remainder is yet being actively surveilled for relapse. Among these models, we have previously described LTL-313B and LTL-331 as models for CRPC and NEPC development, respectively^21, 22^. In both models, surgical Cx resulted in a significant decrease in PSA levels and tumor volumes which remained low for at least 5 weeks. In contrast, the effects are sharply reversed upon relapse wherein a significant increase in tumor volume was observed in both models^21^. Both models demonstrated features of ADT-induced dormancy. To determine the dormant status of tumor cells, cell proliferation and apoptosis were evaluated by immunohistochemical staining for human-specific anti-Ki-67 and anti-Caspase 3 (Casp-3) antibodies, respectively, in the Cx series of LTL-313B PDXs. The hormone-naive tumors demonstrated high levels of Ki67 expression and low levels of Casp-3 expression (Fig. 1C). Immediately upon Cx, low levels of Ki67 and Casp-3 were observed at both mRNA and protein levels in tumor cells, further confirmed with IHC staining. Twelve weeks after Cx, the cell proliferation and apoptosis drastically reduced to a state of cellular inactivity (Supplementary Figure S1B). In the relapsed PDXs, tumor growth rapidly increased and Ki67/Casp-3 levels returned to a similar state prior to Cx. Taken together, these findings indicate that ADT can lead to therapy-induced dormancy and development of relapsed tumors in the PCa PDX models.

### miRNAome profiling of PCa progression in PDX models reveals miR-100-5p’s importance post androgen ablation

Genome-wide miRNA expression profiling was performed on the three stages of Cx series (pre-Cx, post-Cx dormancy, and relapse) for the LTL-313B and the -331 models that recapitulate clinical progression into CRPC and NEPC phenotypes, respectively.

Through analysis of microRNA profiles, we found a total of 277 miRNAs that are expressed in LTL-331 PDX models in all stages of disease from treatment naïve pre-castrate tumors to castration induced dormancy to NEPC relapse phase. Similarly, 319 miRNAs were consistently expressed in all three LTL-313B models with a CRPC relapse fate (Supplementary Table S1). We identified 18 and 19 miRNAs that are not expressed in primary hormone naïve prostate tumors but emerge post therapy (dormancy phase) and in NEPC or CRPC state, respectively. Out of these, 5 miRNAs are common between NEPC and CRPC models, namely, miR-379-5p, 136-5p, 181-5p, 376a-3p, and 219a-5p (Supplementary Table S4). Additionally, we find 30 and 8 miRNAs that are exclusively expressed in dormant phases of NEPC and CRPC, respectively. Out of these, there are 4 miRNAs, namely, miR-31-5p, 486-5p, 144-5p, and 129-3p, common between the two models (Supplementary Table S4).

**Figure 2:**
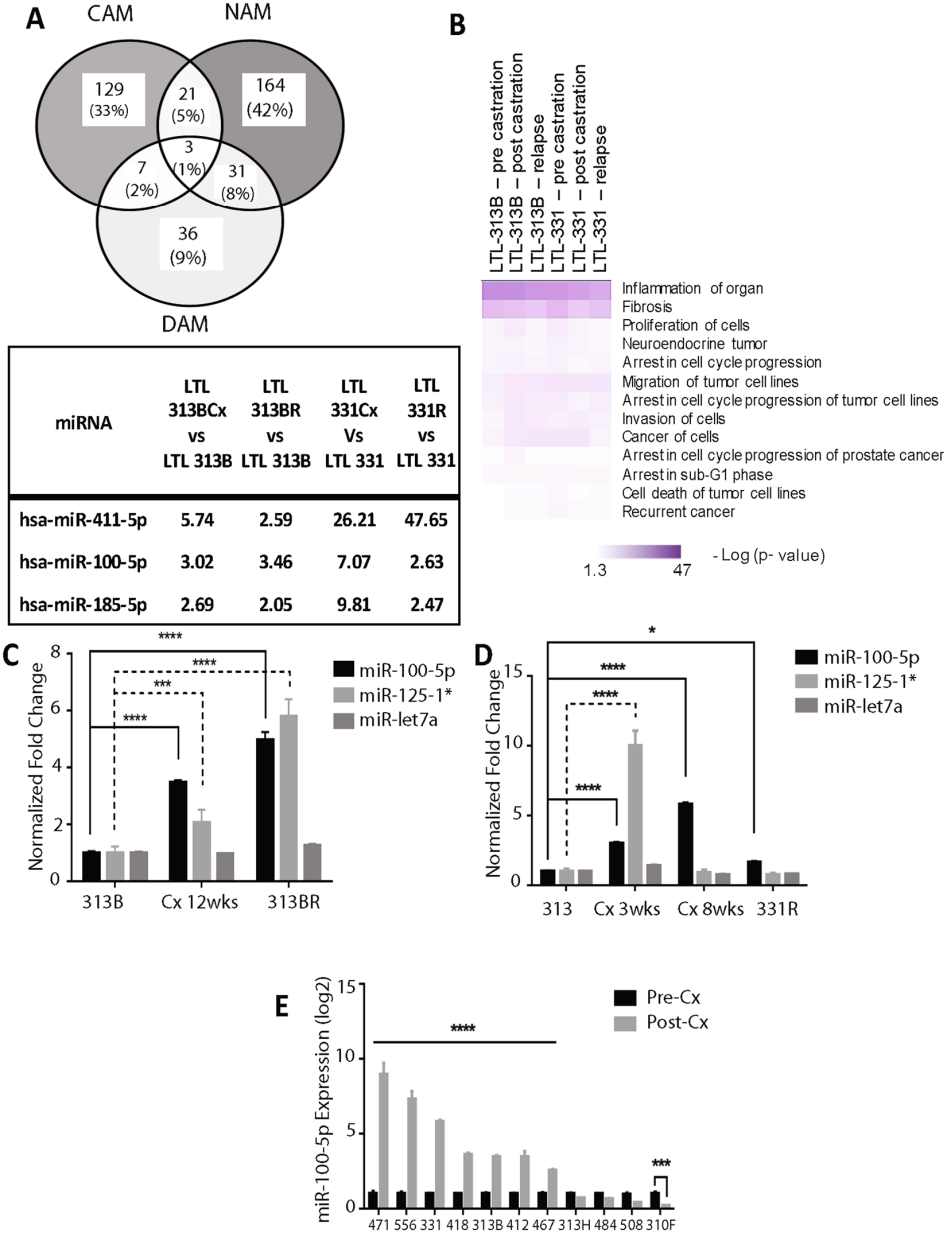
MicroRNAome profiling reveals pathways associated with prostate cancer biology and identifies miR-100-5p association with ADT. (**A**) Identification of candidate miRNAs that were up-regulated during the post-castration period and in both CRPC/NEPC relapses. microRNA-specific microarray was performed on hormone-naive (LTL-313B/-331), post-castrated (LTL-313BCx/-331Cx), and relapse (LTL-313BR/-331R) in PCa PDX progression models for CRPC and NEPC, respectively. microRNAs with a fold change of >2.0 compared to LTL-313B/-331 were identified as being up-regulated for post-castration in both models (dormancy-associated microRNAs; DAM), CRPC relapse (CRPC-associated microRNAs; CAM), or NEPC relapse (NEPC-associated microRNAs; NAM). microRNAs that were specifically up-regulated during the post-castration period and in both relapses were determined by overlapping DAM, CAM, and NAMs. (**B**) Top representative canonical pathways associated with LTL-313B and LTL-331 PDX models. microRNA microarray analysis of hormone sensitive and insensitive PDX models reveals miR-100-5p being consistently expressed, and upregulated in both CRPC (LTL-313B) and NEPC (LTL-331) fated tumors post therapy. (**C**,**D**) miR-100-5p is upregulated *in vivo* upon androgen ablation (host castration) in both CRPC and NEPC fated tumors. Mature microRNA expression from the MIR100HG cluster are also evaluated in LTL-313B/-331 castration (Cx) series (pre-Cx, post-Cx, relapse) via qPCR. (**E**) miR-100-5p expression was measured in 11 representative pre- and post-Cx PCa PDX models via qPCR.

We stratified the samples based on response to ADT (Fig. 2A). miRNAs were considered to be dormancy-associated miRNAs (DAM) if >2.0 fold change was observed during the post-Cx period in both LTL-331 and -313B models. Similarly, miRNAs were considered to be NEPC-associated miRNAs (NAM) or CRPC-associated miRNAs (CAM) if >2.0 fold change was observed in the relapse period of each model, compared to the hormone-naive sub-line. Based on these criteria, the microarray data revealed 35 DAM, 219 NAM and 160 CAM out of a panel of 2548 miRNAs. This analysis identified 3 miRNA candidates upregulated during dormancy period and associated with both CRPC and NEPC relapse. Specifically, miR-100-5p, miR-411-5p, and miR-185-5p, were identified to be associated with ADT. In order to identify the enriched pathways associated with miRNA expression, we used IPA. The top canonical pathways identified from the expressed miRNAs in each phase of both models include inflammation, cell proliferation, arrest in cell cycle progression, invasion, and migration (Fig. 2B).

Although all three microRNAs identified post ADT have been associated with cancer^23, 24^, we focused on miR-100-5p, since it demonstrated with the highest expression upon Cx and showed stable expression in the relapsed LTL-313BR PDXs (Fig. 2C,D).

miR-100 is hosted in the MIR100HG cluster, a long non-coding RNA which also produces let-7 and miR-125b. All three mature miRNAs have been implicated in cancer development and progression^25–27^. To investigate their role in Cx-resistance development, the expressions of all three mature microRNAs were analyzed in the LTL-313B and -331 Cx series by qPCR (Fig. 2C,D). In both models, miR-100-5p was immediately up-regulated by 12 and 8wk post-Cx during which high-expression was maintained through relapse compared to the hormone-naïve state. MiR-100-5p expression was further investigated in other PDX models and analyzed for the pre- and post-Cx period. MiR-100-5p was significantly up-regulated in 7 out of 11 PDX models of post-Cx dormancy period (Fig. 2E). Thus, we sought to further investigate miR-100-5p and its role in PCa dormancy and progression.

### MiR-100-5p inhibition induces cell apoptosis in androgen-deprived PCa cells

**Figure 3:**
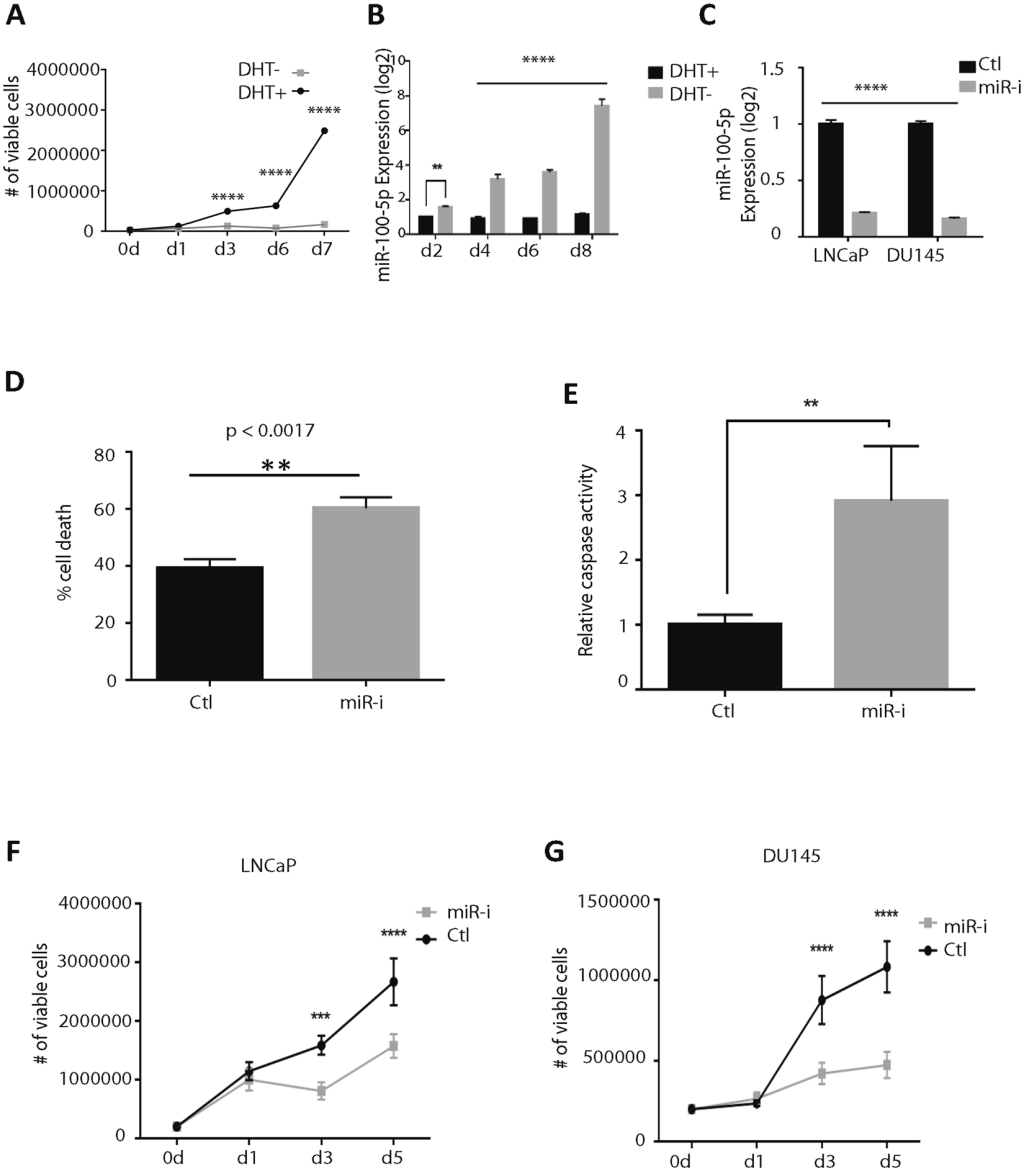
miR-100-5p regulates cell proliferation and apoptosis in prostate cancer cell lines. (**A**) Cell proliferation was assessed by trypan blue count in LNCaP cells grown in either dihydrotestosterone (DHT)-supplemented or –deprived medium (10% CSS) for the indicated time points. (**B**) miR-100-5p expression was measured in DHT-deprived LNCaP cells and assessed via qPCR over the course of 8d. (**C**) miR-100-5p knockdown in LNCAP and DU145 cell lines in the presence of CSS shows an effective reduction in miR-100-5p expression, independent of the AR signaling axis. (**D**) miR-100-5p inhibition in LNCaP cells increases the percentage of cell death as confirmed by trypan blue staining. (**E**) Relative caspase activity measured by caspase 3/7 assay in LNCaP cells shows apoptosis in Cx-resistant adenocarcinoma cells (LNCaP). (**F**–**G**) Number of viable cells as a consequent of miR-100-5p inhibition shows reduction in proliferation in both CRPC (LNCaP) and NEPC (DU145) representative cell lines.

To re-capitulate ADT-induced dormancy *in vitro*, LNCaP cells were subjected to androgen-deprivation using a protocol adapted from Buttyan *et al*.^28^ LNCaP cells are established from a human lymph node metastatic lesion of prostatic adenocarcinoma, express AR, and are dependent on AR signaling for proliferation. Androgen-deprived LNCaP cells were marked by a significant reduction in cell proliferation (Fig. 3A) with no visible signs of apoptosis. These results supported the observations made by Buttyan *et al*.^29^ and provided a rationale for use of LNCaP cells as an *in vitro* model for ADT-induced prostate cancer dormancy. The expression of miR-100-5p was then assessed in androgen-deprived AR^+^ LNCaP cells by qPCR analysis in the presence or absence of androgen, to mimic the LTL-313B PDX model *in vitro*. Upon androgen-deprivation, miR-100-5p expression was dramatically up-regulated over the course of 8 days (Fig. 3B). Interestingly, and in keeping with PDX lines, miR-100-5p was expressed in both AR^+^ LNCaP and AR^−^ DU145 cells (Fig. 3C). Since androgen-deprivation induced miR-100-5p up-regulation in both prostate PDX and *in vitro* models for ADT-induced PCa dormancy, we proceeded to study the effects of manipulating miR-100-5p expression *in vitro*. To this end, LNCaP cells were subjected to androgen-deprivation (CSS treatment) for 5 days along with miR-100-5p inhibition or control. Trypan blue counts demonstrated significant cell death in androgen-deprived, miR-100-5p-silenced cells (Fig. 3D); moreover, caspase 3/7 assay showed that miR-100-5p inhibition induced cell apoptosis in androgen-deprived LNCaP cells (Fig. 3E). Together, these two findings suggest a critical role of miR-100-5p in cell survival upon androgen deprivation of AR^+^ PCa cells.

We then set out to identify the role of miR-100 in proliferating cancer cells. Interestingly, in both LNCaP and DU145 cells grown in normal conditions, miR-100-5p inhibition significantly reduced cell proliferation (Fig. 3F,G). Taken together, these results indicate that miR-100-5p is required for the proliferation of AR^+^ and AR^−^ PCa cells, and for the survival of dormant PCa cells exposed to ADT.

### Molecular targets of miR-100-5p and their implicated enriched pathways

Using TargetScan (Release 7.1)^30^, we found 60 genes are predicted to be a target of miR-100-5p miRNA (Supplementary Table S2). We further consulted with miRDB and TarBase (Release version 7.0) databases for finding highly enriched predicted genes and found 27 and 583 predicted genes for miR-100-5p, respectively (Supplementary Table S2). Supplementary Table 3 provides all enriched KEGG and GO canonical pathways as well as predicted upstream regulators.

**Figure 4:**
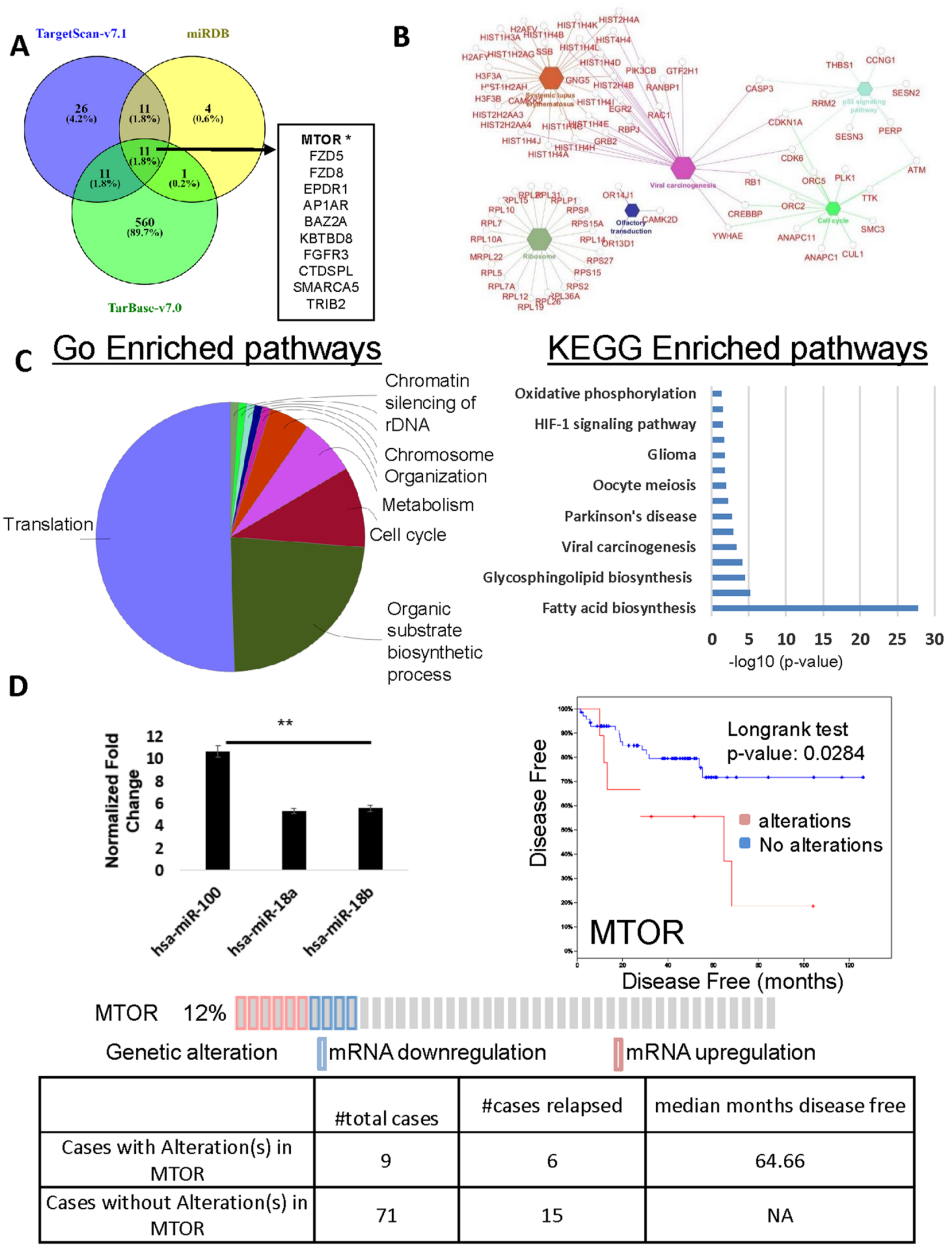
Curated and predicted interaction network for miR-100-5p. (**A**) Three databases were used to find predicted gene and protein interactions with miR-100-5p. (**B**) Enriched canonical pathways using KEGG and GO pathway databases for predicted targets of miR-100-5p. Octagons represent major canonical pathways and associated genes, in red, are presented in round nodes circulating the pathways (generated using Cytoscape). (**C**) Gene networks regulated by miR-100-5p based on canonical pathways from GO and KEGG enrichment analyses associated with genes that are predicted to be regulated by miR-100-5p. (**D**) miR-100-5p expression in Taylor *et al*.^32^ MSKCC database finds a significant correlation between poor survival and alterations in target of miR-100-5p, MTOR.

The miR-100-5p-regulated putative targets associated with human disease were predicted using Ingenuity Pathway Analysis (IPA) and Cytoscape v3.4.0 (ClueGo v2.3.2 application) softwares (Fig. 4B). We found that cancer and cancer-associated pathways are deeply linked to miR-100-5p’s predicted targets (Fig. 4C). This is in keeping with earlier studies reflecting miR-100-5p’s involvement in an extensive range of cancer types. Moreover, molecular and cellular functions included “cellular growth and proliferation” and “cell cycle” both of which have previously been linked to aberrant miR-100-5p expression in medical literature^23, 31^. This is further corroborated in the miR-100-5p target gene list for proliferation CDKN1A, CDK6, RB1 and apoptosis ATM and CASP3. Canonical pathways of downstream targeted genes regulated by miR-100-5p include HIF1-a, P53, VEGF, NRF2-mediated oxidative stress, EGF, and EFG signaling pathways among others.

Interestingly, we found that MTOR is one of the targets of miR-100-5p as predicted by all three databases (Fig. 4A). According to the microRNA target database (2010 release), miR-100-5p binds to position 281 nucleotide sequence of MTOR with a predicted mirSVR score of −1.1698. These machine-learning algorithms ranked microRNA target sites by likelihood of downregulation from sequence and structural features of microRNA/mRNA predicted target sites. Specifically, the predicted network of experimentally validated miR-100-5p target interactions is generated with TarBase database (Supplementary Figure S2) and shows that the interaction of MTOR and miR-100-5p are particularly strong. The mirSVR cutoff is less than or equal to −1.2 for top 5% probability of binding.

We further queried a publicly available clinical cohort of 216 PCa adenocarcinoma patient samples^32^. Unsurprisingly, miR-100-5p was universally expressed in all of the patient samples, and its median expression was higher than well-characterized oncogenic microRNAs (Fig. 4D). In addition, the highest ranking miR-100-5p target, (MTOR), a favorable prognostic factor in PCa^33^, was significantly associated with longer disease-free survival, implicating the clinical significance of mir-100-5p targets.

Experimentally, we found that miR-100 overexpression for 72 hours decreases MTOR protein expression levels in LNCaP cells (Supplementary Figure S3), consistent with data in smooth muscle cells^34^.

## Discussion

PCa can often escape therapeutic pressure by entering a dormant state and subsequently by transforming into an incurable neuroendocrine (NEPC) or castration resistant (CRPC) disease. In this study, we identified critical microRNAs that drive these processes, exploiting our unique and previously well-characterized PCa PDX models. Specifically, we used microRNA profiling of these tumor lines to investigate the process through which prostate adenocarcinomas transition to therapy resistant malignant tumors. Our findings indicate that, while some microRNAs are transiently upregulated during one stage the process, miR-100-5p is persistently upregulated, and is involved in dormancy, as well as in NEPC and CRPC progression. The association of inflammation, survival, and invasion pathways with microRNAs are particularly interesting and largely unexplored in PCa and pose as a stepping stone for further investigations.

The growing importance of miR-100-5p in carcinogenesis has been realized by several groups, which have investigated the multifaceted role of this microRNA in numerous cancers^35^. More specifically, miR-100 has been shown to regulate apoptosis and cell cycle arrest through regulating myotubularin related protein 3 (MTMR3) in breast cancer cells^36^. MTMR3 inhibition through miR-100 activates p27 that can either turn on apoptosis through Bax/Bcl-2 or G2/M cell cycle arrest through CDK1/Cyclin B. An analysis of human PCa samples by Leite *et al*. revealed a global de-regulation of miR-100-5p during the transition from high grade localized PCa to metastasis^37^. Loss of miR-100-5p was concurrently reported to promote PCa metastasis through regulation of migration, invasion, EMT, and stemness of cancer cells by up-regulating AGO2 expression^38^. In sharp contrast, however, patients with higher levels of miR-100-5p were more likely to relapse following radical prostatectomy^39^. In light of these studies, Leite *et al*. suggested a context-dependent role for miR-100-5p in PCa, which heavily depends on the stochastic needs of a tumour cell upon therapeutic insults and nutrient availability. Oncogenic miR-100-5p can stimulate rapid proliferation by down-regulating SMARCA5, BAZ2A, and THAP2 in the pRb signalling pathway. In a reversed role, miR-100-5p could potentially down-regulate mTOR pathway thus reducing cell proliferation^40^. Further, our data indicates that miR-100 is induced by androgen ablation and concordantly androgens inhibit miR-100 expression. Even though we do not explore how miR-100 expression is regulated, we can infer that miR-100-5p plays a double role in promoting hormone resistance: it is acutely up-regulated upon ADT and therefore it is required for androgen-independent survival in dormant PCa cells. To the contrary, in AR-negative cells, miR-100 promotes cell proliferation. In keeping with this multifaceted role, our clinical and bio-informatics data suggest that miR-100-5p inhibits the expression of several other tumor-suppressive genes, which are involved in both androgen-independent survival and proliferation.

Taken these results and previous publications suggest that miR-100-5p expression may play a double role in PCa: it is required to maintain the dormant state of PCa cells acutely exposed to ADT; however, miR-100-5p expression is also essential in retaining proliferative and tumorigenic capacity through appropriate modulation of miR-100-5p targets and can act as a novel driver and potential therapeutic target for preventing the emergence of CRPC and NEPC. As such, our investigation highlights a novel framework in identifying dormancy-associated miRNAs, and to reveal their therapeutic potential.

## Materials and Methods

### Patient-Derived Xenograft (PDX) PCa Mouse Models

Twelve patient-derived PCa mouse models have been generated at the Living Tumor Laboratory (LTL, http://livingtumorcentre.com, Vancouver, BC, Canada), as described previously^21^. Specimens were obtained from patients following a protocol approved by the Clinical Research Ethics Board of the University of British Columbia (UBC) and the BC Cancer Agency. All experimental protocols for animal model generation have been approved by UBC’s licensing committee and in accordance with institutional guidelines and regulations. The specimens were examined, sectioned and selected by pathologists for histological analysis and xenografting. All patients signed a consent form approved by the Ethics Board as well as department and institutional boards (UBC Ethics board #: H09-01628 and H04-60131; VCHRI #: V09-0320 and V07-0058) and permitting publication of anonymized associated information. No tissues were procured from prisoners. In brief, PCa biospecimens from patient surgeries or biopsies are proprietarily grafted into the subrenal capsule of NOD-SCID mice. Upon engraftment and sustained maintenance, the models are subjected to androgen ablation and actively surveilled for tumor volume, PSA, and metastatic relapse. More specifically, LTL-331 relapses as NEPC post androgen deprivation therapy while LTL-313B relapses as CRPC. The fate of other PDX models is depicted in Supplementary Figure S1.

### Cell Culture Experiments

Two human PCa cell lines (LNCaP and DU145) were used to mimic CRPC and NEPC PCa phenotypes *in vitro*. Cells were maintained in RPMI-1640 supplemented with 10% FBS or charcoal stripped serum (CSS) in a humidified incubator at 37 °C and 5% CO2. CSS depletes the cells from androgen to mimic clinical ADT treatment. For cell count experiments, cells were trypsinized to form a single-cell suspension and counted using a TC20 Automated Cell Counter (Bio-Rad). Transfections were carried out in Opti-MEM with Lipofectamine RNAiMAX (Invitrogen, Carlsbad, CA, USA), diluted 1:100 according to the manufacturer’s protocol. *AntimiR-100-5p for mature miR-100-5p (* AACCCGUAGAUCCGAACUUGUG) (catalog number AM17000) and control *antimiR* (catalog number AM17010) were purchased from Ambion (Austin, TX, USA). Cells were transfected in 60mm dishes with inhibitors at 30 nm for 48 hours (unless otherwise indicated). Cells were trypsinized with 0.25% EDTA-trypsin (ThermoFisher, Cat# 25200072), and lysed in 80–100 μL of RIPA buffer. Samples were loaded on 10% SDS gel at 50ug in 20uL, run at 100 V for 1 hr, and transferred to nitrocellulose membranes at 100 V for 1.25 hr. 5% BSA in Tris- Buffered Saline containing 0.1% Tween (TBST) was used to block membranes and incubate antibodies. Membranes were incubated in primary antibody solutions for β-actin at 1:5,000 (Sigma, Cat# A5441) and MTOR at 1:1,000 (Cell Signaling, Cat #2972) overnight at 4 degrees celcius. They were then washed 3x in TBST for 5 minutes and incubated with HRP-conjugated anti-mouse (B-actin) and anti-rabbit (MTOR) antibodies (1:5,000) for 1 hour. After washing as above, membranes were developed with chemiluminescence a GelDoc system using an ECL western blotting substrate kit (ThermoFisher, Cat# 32016). Caspase 3/7 Assay in cell lines. After 24 h androgen deprivation, 2,000 LNCaP cells were seeded into a 96-well plate and transfection was performed the next day. Upon 5d incubation, Caspase 3/7 assay was carried out according to the manufacturer’s protocol.

### mRNA and microRNAome Expression Microarrays

mRNA and microRNA expression profiling was done using the services provided by Laboratory for Advanced Genome Analysis (Vancouver, BC Canada). For gene expression profiling, total RNA samples were prepared following Agilent’s One-Color Microarray-Based Gene Expression Analysis Low Input Quick Amp Labeling v6.0 as previously described (24356420). Samples were hybridized on Agilent SurePrint G3 Human GE 8 × 60 K Microarray (AMDID 028004). microRNA expression- profiling was performed by Cyanine-3 end-labeling of microRNA following Agilent’s microRNA Microarray System with microRNA Complete Labeling and Hyb Kit v3.1.1 and hybridization with Human microRNA Microarray Release 21.0 (AMDID 070156). Arrays were scanned with the Agilent DNA Microarray Scanner at a 3um scan resolution and data was processed with Agilent Feature Extraction 11.0.1.1. Processed signal was quantile normalized with Agilent GeneSpring 12.0. The log2 transformed expression level (FC > 2.0) of CRPC, NEPC, and dormancy-associated microRNAs are presented in the supplementary tables.

### Immunohistochemical Staining of PDX Tumors

Formalin-fixed paraffin-embedded tumor tissues were sectioned, probed, and stained with DAB (Sigma) as previously described. (16155594). IHC staining was performed using mouse (Dako, Mississauga, Ontario) and rabbit (Cell Signaling, Danvers, Massachusetts) polyclonal antibodies against human Ki67 and Casp-3, respectively (1:50 dilution).

For quantification of immunohistochemical staining of cells by Ki-67 and Caspase-3, ten randomly selected high-power images from each PDXs were captured using an AxioCam 506 Color mounted on an Axioplan 2 microscope and a Zen image software (Carl Zeiss), with final magnifications of ×400. Ki-67– or Caspase-3–positive cells and total cells (positive + negative) were counted separately. Numbers of Ki-67– or Caspase-3–positive cells per 1,000 tumor cells (Ki-67 or Caspase-3 index) were calculated using the formula: Index = number of positive cells × 1000/number of total cells.

### Quantitative Real-Time PCR Experiments

Cell pellets were collected using 0.25% EDTA-trypsin (ThermoFisher, Cat# 25200072) prior to RNA extraction. Total RNA, containing small RNAs, was isolated with the Ambion mirVana kit (Ambion, AM1560, Austin, TX, USA) at 4 degrees celcius as recommended by the manufacturer. Quantitative PCR of *miR-100-5p*, miR-125b-1*, and *miR-let7a* was performed with TaqMan assays from Applied Biosystems (Foster City, CA, USA) with nuclease-free water (Ambion, Cat#: AM9937), Taqman Universal Master Mix II with UNG (ThermoFisher, Cat# 4440038), and gene-directed Taqman probes (ThermoFisher). 10 ng RNA that was treated with DNAse and reverse transcribed using primers and MultiScribe reverse transcriptase (Applied Biosystems, Foster City, CA, USA). Samples were amplified in a 7900HT real-time PCR system (Applied Biosystems, Foster City, CA, USA). Gene expression was calculated through the 2-ΔΔCt method and normalized to the mir-30e (*in vivo*) and miR-130b (*in vitro*), which have been characterized as reliable reference genes by our group^41^ and by independent investigators^42^.

### Computational Bioinformatics Analyses

microRNA expression data were first log2 transformed. Hierarchical clustering was performed with average linkage and Pearson correlation. Before clustering, the up and down-regulated miRNAs were filtered to eliminate genes with no differential expression among samples. Statistical analysis was performed using GraphPad Prism 6 (GraphPad Software, Inc). The Student *t* test was carried out to compare means between two groups. One-way ANOVA followed by the *post hoc* Dunnett test was used to compare means of more than two groups. We used previously published data from MSKCC clinical cohort^32^ for survival analyses.

### Ethics

All methods, including mouse model generation, were carried out in accordance with University of British Columbia’s guidelines and regulations and all experimental protocols were approved by University of British Columbia’s licensing committee.

## Electronic supplementary material

Supplementary Figures

Supplementary Table S1

Supplementary Table S2

Supplementary Table S3

Supplementary Table S4
