# Supplementary material for: miR-100-5p inhibition induces apoptosis in dormant prostate cancer cells and prevents the emergence of castration-resistant prostate cancer
Source: Sci Rep. 2017 Jun 22;7:4079. doi: 10.1038/s41598-017-03731-8 (PMC5481412; doi:10.1038/s41598-017-03731-8)
Supplement: Supplementary file 1 — Supplementary Figures [file 41598_2017_3731_MOESM1_ESM.pdf]

miR-100-5p inhibition induces apoptosis in dormant prostate cancer cells and prevents the emergence of castration-resistant prostate cancer

Noushin Nabavi[2,3,5*], Nur Ridzwan Nur Saidy[1,2*], Erik Venalainen, Anne Haegert[3], Abhijit Parolia[1,2], Hui Xue[2,3], Yuwei Wang[2,3], Rebecca Wu[2,3], Xin Dong[2,3], Colin Collins[3], Francesco Crea[2,4**] Yuzhuo Wang[** 2,3,5]

Figure S1

A

**Tumor Line**	**Histopathology**	**AR/PSA Status (+/-)**	**Relapse (Y/N/UND)**	**Tumor Sub-Line**	**Histopathology**	**AR/PSA Status (+/-)**
LTL-310F	ADC	+/+	Y	LTL-310FR	CRPC	+/+
LTL-311C	ADC	+/+	Y	LTL-311CR	CRPC	+/+
LTL-313B	ADC	+/+	Y	LTL-313BR	CRPC	+/+
LTL-313H	ADC	+/+	Y	LTL-313HR	CRPC	+/+
LTL-418	ADC	+/+	Y	LTL-418R	CRPC	+/+
LTL-484	ADC	+/+	Y	LTL-484R	CRPC	+/+
LTL-331	ADC	+/+	Y	LTL-331R	NEPC	-/-
LTL-412	ADC	+/+	UND			
LTL-467	ADC	+/+	UND			
LTL-471	ADC	+/+	UND			
LTL-508	ADC	+/+	UND			
LTL-556	ADC	+/+	UND			

B

**LTL PDX Models**	**Ki67 Fold change**	**Ki67 Fold change**
LTL-313B Post-Cx/ Pre-Cx	-1.99	0.99
LTL-556 Post-Cx/ Pre-Cx	-1.15	0.96
LTL-313H Post-Cx/ Pre-Cx	-1.49	1.02
LTL-412 Post-Cx/ Pre-Cx	-1.10	1.01
LTL-471 Post-Cx/ Pre-Cx	-1.76	0.92
LTL-310F -Post-Cx/ Pre-Cx	-1.69	0.92
LTL-467 Post-Cx/ Pre-Cx	-0.30	0.96
LTL-331 Post-Cx/ Pre-Cx	0.17	0.99
LTL-418 Post-Cx/ Pre-Cx	-2.71	0.98
LTL-484 Post-Cx/ Pre-Cx	0.85	0.97
LTL-508 Post-Cx/ Pre-Cx	-0.48	0.92

Supplementary Figure S1. (A) Characterization of PCa PDX models for CRPC and NEPC relapse. Twelve hormone-sensitive prostate PDX models were obtained and subjected to surgical castration (androgen deprivation). Upon the emergence of a castration-resistant sub-line, pathological examination was stratified into either the adenocarcinoma (CRPC) or neuroendocrine (NEPC) phenotype (abbreviations: LTL=living tumor laboratory, ADC=adenocarcinoma, Y=yes, N=no, UND=undetermined, CRPC=castration-resistant prostate cancer, NEPC=neuroendocrine prostate cancer)**.** (B) Characterization of tumor dormancy based on Ki67 and Casp-3 mRNA expression levels. The log2 transformed mRNA expression fold change was calculated for pre-castrate compared to dormant post-castrate PDX tumors (total of 11 tumors). Markers of proliferation (Ki67) and apoptosis (Casp-3) are both downregulated in the post-cx dormant tumors compared to untreated pre-castrate tumors.

miR-100-5p inhibition induces apoptosis in dormant prostate cancer cells and prevents the emergence of castration-resistant prostate cancer

Noushin Nabavi[2,3,5*], Nur Ridzwan Nur Saidy[1,2*], Erik Venalainen, Anne Haegert[3], Abhijit Parolia[1,2], Hui Xue[2,3], Yuwei Wang[2,3], Rebecca Wu[2,3], Xin Dong[2,3], Colin Collins[3], Francesco Crea[2,4**] Yuzhuo Wang[** 2,3,5]

Figure S2

A

B

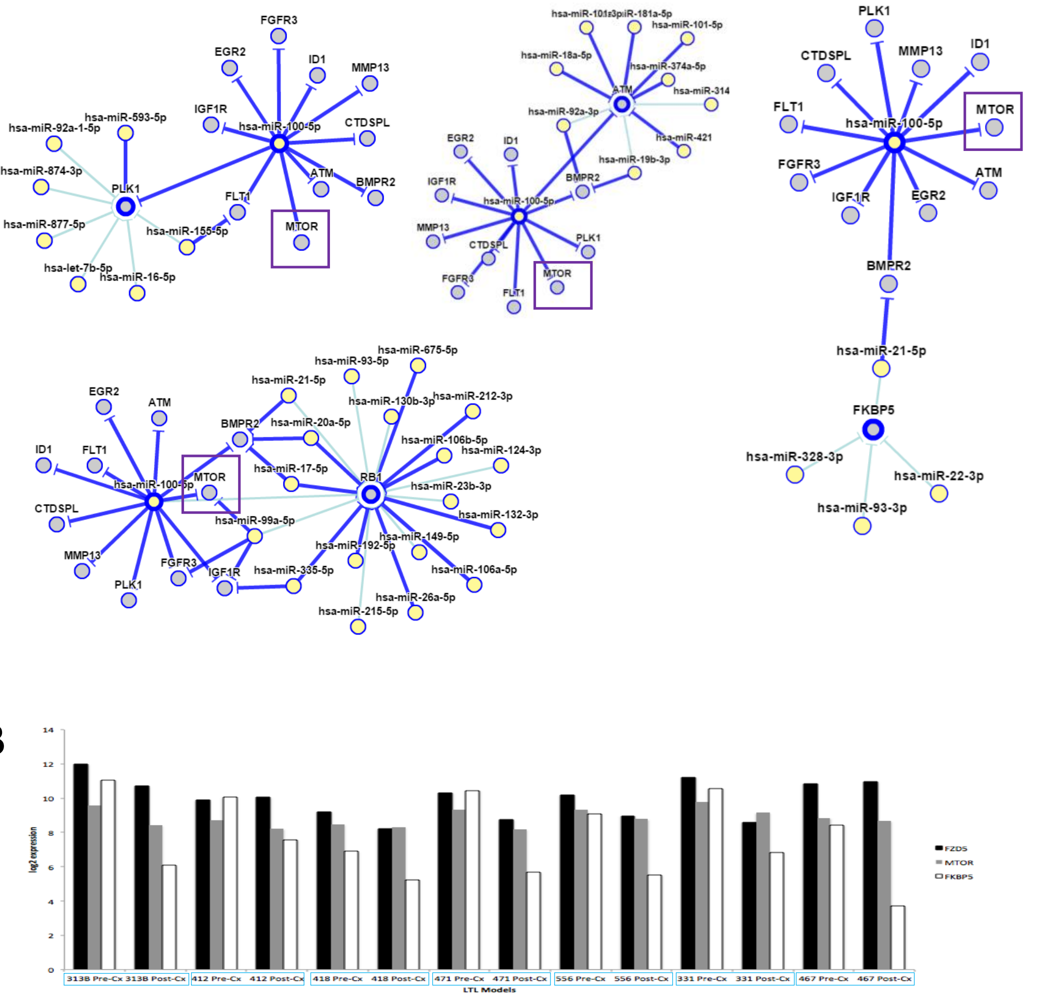

Supplementary Figure S2. Curated and predicted interaction network for miR-100-5p. (A) Predicted integrative interaction network for miR-100-5p from TarBase, including mTOR. Dark blue lines represent a strong evidence for interaction between nodes confirmed by reporter assays, western blots, qRT-PCR or qPCR and light blue represents other evidences. (B) *FZD5, MTOR, FKBP5* expression was measured in 11 pre- and post-Cx PCa PDX models via qPCR.

Noushin Nabavi[2,3,5*], Nur Ridzwan Nur Saidy[1,2*], Erik Venalainen, Anne Haegert[3], Abhijit Parolia[1,2], Hui Xue[2,3], Yuwei Wang[2,3], Rebecca Wu[2,3], Xin Dong[2,3], Colin Collins[3], Francesco Crea[2,4**] Yuzhuo Wang[** 2,3,5]

Figure S3

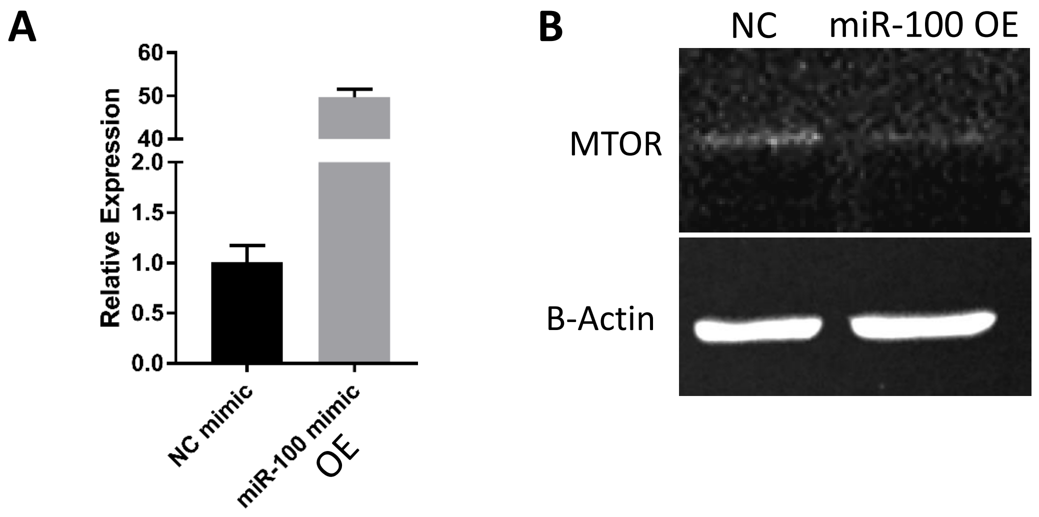

Supplementary Figure S3. miR-100-5p regulation of MTOR in LNCaP cell line. (A) Relative qPCR expression of miR-100-5p following overexpression (OE), compared to negative control (NC). miR-100-5p was overexpressed upon 30nm of miR-100 mimic treatment for 72 hours in LNCaP cells. (B) Western blots showing MTOR protein levels are lowered following miR-100 OE after 72 hours of treatment, B-actin is used as loading control.
